# Profile of Genes Encoding Antimicrobial Resistance and Virulence in 3963 *Escherichia coli* Isolates

**DOI:** 10.1155/bmri/9411547

**Published:** 2026-11-04

**Authors:** Debyani Samantray, Ankit Singh Tanwar, Arya Sudheer, Infant Thomas, Abhishek B. Kannur, Thokur Sreepathy Murali, Ashma Dorothy Monteiro, Shivakumara Siddaramappa, Bobby Paul

**Affiliations:** ^1^ Department of Bioinformatics Manipal School of Life Sciences Manipal Academy of Higher Education Manipal India manipal.edu; ^2^ Department of Circulation and Medical Imaging Norwegian University of Science and Technology – NTNU Trondheim Norway; ^3^ Department of Public Health Genomics Manipal School of Life Sciences Manipal Academy of Higher Education Manipal India manipal.edu; ^4^ Department of Data Science Prasanna School of Public Health Manipal Academy of Higher Education Manipal India manipal.edu; ^5^ Prokaryotic Research Consortium of Karnataka Ganeshana Beedhi Karnataka India

**Keywords:** antibiotic resistance genes, bacterial toxin, intracellular gene transfer, multidrug resistance, pangenome analysis

## Abstract

The prevalence and spread of antimicrobial resistance genes (ARGs) and virulence‐associated genes (VAGs) in bacterial pathogens is a major public health burden. *Escherichia coli* is an opportunistic pathogen that has garnered significant attention because of the emergence and dissemination of resistance to antibiotics of human and veterinary importance. The objective of this study was to characterize ARGs and VAGs in 3963 *E. coli* genomes released in complete level by the NCBI from 2006 to 2025. A total of 293 distinct ARGs and 351 distinct VAGs were identified from the genomes of *E. coli* isolates. Among the 293 ARGs, presence of 41 were found in the genomes of *E. coli* strains submitted since 2006, and the remaining 252 ARGs were identified in the genomes submitted in subsequent years. The genomes were enriched with ARGs corresponding to the drug classes fluoroquinolones, penam, tetracyclines, cephalosporins, and macrolides. The analysis identified that in 2037 (51.4%) isolates ARGs represented at least 1.29% of the genome, and in 1987 (50.13%) isolates accounted for at least 1.35% of their genome for virulence genes. Analysis of the distinct ARGs showed that 123 of them were found only on plasmids and 141 were shared between chromosomes and plasmids, whereas VAGs showed a greater tendency for chromosomal localization. Single‐copy occurrences of *crp* and *cpxA* were commonly found across all 3963 isolates, whereas multiple‐copy variants (≥ 2 copies) of *sul1*, *tet(A)*, and *bla_TEM-1_* were detected in approximately 5% of the isolates. Sequence type analysis showed that ST10 was most frequently represented sequence type in higher level ARG Group III and IV, whereas almost all the strain in ST11 and ST73 were harbouring higher level of Group IV virulence. Weighted linear regression indicated an increase of 0.00292 percentage points per year in the genomic proportion encoding ARGs in *E. coli*. The results suggest a need for continuous genomic surveillance to tackle AMR, and the development of alternative approaches to overcome the burden of AMR and virulence.

## 1. Introduction

Antimicrobial resistance (AMR) is a leading public health concern, as it threatens the effectiveness of antibiotics and contributes substantially to the morbidity and mortality associated with bacterial infections [1–3]. The World Health Organization (WHO) has emphasized that AMR threatens the effective prevention and treatment of an increasing range of infections [4]. A recent study using predictive statistical models reported that 4.95 million deaths were associated with bacterial AMR in 2019, including approximately 1.27 million deaths attributable to bacterial AMR [5]. Additionally, the mortality associated with AMR is greater than that caused by HIV‐AIDS or malaria [5]. *Escherichia coli* is a clinically important bacterium and is a common cause of urinary tract, bloodstream, and gastrointestinal infections. It has been reported that *E. coli* is among the six leading pathogens associated with increased mortality worldwide [5], and antimicrobial resistant strains can further reduce treatment options [6–8]. Advances in sequencing technologies have led to the availability of many bacterial genomes in public databases, providing valuable resources for investigating the genomic basis and distribution of AMR [9–13]. Whole‐genome data analysis with sophisticated bioinformatics resources can provide insight into the prevalence and distribution of AMR and virulence‐associated genes (VAGs).

Bacteria can become resistant to antibiotics through various mechanisms [14, 15], such as (i) limiting the uptake of drugs, (ii) modifying drug targets, (iii) inactivating drugs, and (iv) promoting active drug efflux. Bacterial pathogens are also known to contain genetic elements that confer resistance against antimicrobial agents. Mobile genetic elements contribute to dissemination of ARGs through different mechanisms. Plasmids are independently replicating DNA molecules that can carry multiple ARGs, whereas transposons and insertion sequences can mobilize genetic regions within or between DNA molecules. Integrons can capture and organize gene cassettes, including resistance determinants, and may occur on chromosomes or plasmids. Together, these elements can facilitate the movement and maintenance of ARGs within bacterial populations [16]. Plasmids can carry multiple antimicrobial resistance genes (ARGs) and contribute to their dissemination among bacterial populations [17]. Multidrug resistance plasmids can contribute substantially to dissemination of ARG, and some ARGs are predominantly detected on plasmids [18]. In addition, ARGs can be exchanged intracellularly between chromosomes and plasmids [16]. However, a study reported that the transfer of ARGs between plasmids and chromosomes is a rare event [19].

The identification and analysis of ARGs associated with plasmids can offer insights into mobile genetic elements and plasmid‐mediated AMR [20]. Tadesse et al. [21] characterized 1729 *E. coli* strains isolated from humans and animals from the year 1950 to 2002 and identified carriage of ARGs conferring resistance to ampicillin, sulfonamide, and tetracycline. In another study, 100 *E. coli* strains isolated from neonates were found to be resistant to amoxicillin, cefuroxime, and ceftriaxone [22]. However, the presence of ARGs and VAGs in bacterial chromosomes and plasmids, especially in different *E. coli* strains, has not been studied in a longitudinal time frame. Furthermore, the genomic constitution of ARGs and VAGs in various *E. coli* isolates remains understudied. The objective of this study was to characterize ARGs and VAGs in the genomes of *E. coli* isolates submitted to the NCBI between 2006 and 2025 that meet the study inclusion and exclusion criteria.

## 2. Materials and Methods

### 2.1. Resistance and Virulence Gene Prediction

Whole‐genome data at the genome database (https://www.ncbi.nlm.nih.gov/genome/) was utilized to estimate the prevalence and distribution of ARGs and VAGs in *E. coli*. A search for query “*Escherichia coli* [Organism]” in the NCBI database showed 376,058 whole genomes that were submitted until 2025. However, only 4446 genomes were released between 2002 and 2025 in “complete” assembly status. Several instances of genome contamination, misassembly of genomes, and reclassification of strains or species of genomes in the public repositories have been reported in the recent past [23]. Draft assemblies may contain unresolved or fragmented genomic regions that can limit reliable chromosome–plasmid localization [24]; therefore, genomes in complete assembly status were only used in the present study. Along with the metadata, whole genome sequences were retrieved in FASTA file format with the help of scripts provided in the Entrezpy library [25] and customized Python scripts. Based on the metadata information, the study included only assemblies with CheckM completeness of ≥ 98%, contamination < 3%, and with NCBI ANI Check status “OK”. Chromosomes of 3978 isolates and strains harboring plasmids were retrieved separately for downstream analysis. Species‐level consistency was further validated by comparing the average nucleotide identity (ANI) with the reference *E. coli* genome using FastANI v1.34 [26]. Downloaded genomes were compared with the reference genome of the *E. coli* strain K‐12 substrain MG1655 (Acc. No. GCF_000005845.2) submitted in 2013. Genomes with ANI values < 96.5% were excluded as likely misclassified genome assemblies [27]. A total of 3963 high‐quality *E. coli* genomes were thus selected for the characterization of ARGs and VAGs based on these inclusion and exclusion criteria. Resistance genes were identified with the help of ABRicate v1.0.1 tool (https://github.com/tseemann/abricate) with the CARD database (January 14, 2025 update) that includes 2631 ARG sequences used to predict the ARGs [28]. Similarly, virulence genes were identified with the help of ABRicate v1.0.1 with the VFDB nucleotide database (January 14, 2025 update) that included 2597 VAGs [29]. The accession numbers of the genomes included in this study are provided in Table S1, and the workflow is summarized in Figure 1.

**Figure 1:**
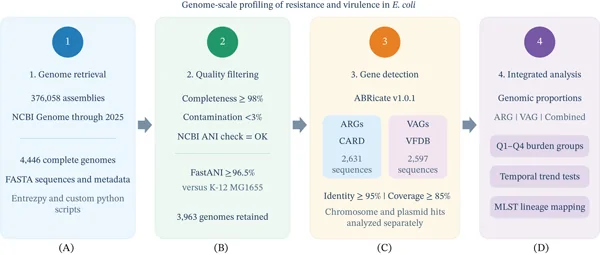
Overview of the workflow employed for the genome‐wide profiling of resistance and virulence in *E. coli*. The study design comprised four major components: (A) whole genome data retrieval, (B) genome quality check and filtering, (C) resistance and virulence gene identification, and (D) integrated ARG‐VAG analysis and visualization.

### 2.2. Estimation of the Genome Constituting for ARGs and VAGs

The genome coordinates for the ARGs and VAGs with ≥ 95% sequence identity and ≥ 85% query coverage were retrieved in CSV file format to perform downstream analyses. The ARGs and VAGs from plasmids and chromosomes were analyzed separately. The copy number of ARGs was inferred from distinct ABRicate CARD hits in the assembled genome sequences. A custom Python script was used to assess genomic coordinates for exact duplicate and overlapping same‐gene hits, though no exact duplicate or overlapping same‐gene hits were identified. The percentages of the *E. coli* genome encoding ARGs and VAGs were estimated. The formula used to estimate the percentage of ARGs and VAGs per genome is provided below:

Percentage=Total size of ARGs or VAGs bpGenome size bp×100.

To avoid arbitrary threshold selection, a data‐driven, quantile‐based (Q1–Q4) stratification approach was applied using the R statistical package (Version 4.5), and to categorize the genomes based on their percentage of ARGs and VAGs encoded. This approach allows partitioning of the dataset based on intrinsic distribution, ensuring that group boundaries are defined by empirical data rather than predefined biological assumptions. Genomes were categorized into four ARG burden groups: Group I (0.98%–1.15%; below 25th percentile), Group II (1.16%–1.28%; between Q1 and the median), Group III (1.2%–1.38%; between the median and Q3), and Group IV (1.39%–1.99%; above 75th percentile). Similarly, VAG burden was grouped as Group I (0.39%–0.83%; below 25th percentile), Group II (0.84%–1.34%; between Q1 and the median), Group III (1.35%–1.83%; between the median and Q3), and Group IV (1.84%–2.87%; above 75th percentile).

### 2.3. Multilocus Sequence Typing (MLST)

Sequence types (STs) were identified using the MLST module at the GitHub portal https://github.com/tseemann/mlst (v2.35.0). Each genome was assigned to specific STs based on the established MLST scheme [30] and further analyzed to identify ARGs and VAGs associated with different STs.

### 2.4. Trend Analysis of ARGs and VAGs

Temporal trends in ARGs, VAGs, and their combined proportions were evaluated across 3963 *E. coli* genomes. Genomes were stratified according to their NCBI assembly submission year, and the mean proportion per year was calculated separately for ARGs, VAGs, and combined ARGs–VAGs. The presence and direction of monotonic temporal trends were assessed using the nonparametric Mann–Kendall test, whereas Sen′s slope estimator was used to quantify the median rate of change over the years. Weighted linear regression analyses were performed to estimate the temporal changes in resistance and virulence genes. The regression coefficient (*β*), 95% confidence interval, coefficient of determination (*R* ^2^), and corresponding *p* value were recorded. All the statistical tests were two‐sided, and a *p* value < 0.05 was considered statistically significant.

### 2.5. Plots and Graphs

All data processing, statistical analyses, and visualizations were performed with the help of RStudio (Version 4.3.1). The R packages such as dplyr, tidyr, stringr, readxl, openxlsx, ggplot2, and heatmap were used for data analyses and to plot the graphs. Violin plot embedded with boxplot was generated to represent the genome size, abundance of ARGs, and VAGs among 3963 *E. coli* isolates. Sankey plot was used to group (Group I–IV) the isolates based on the percentage of genome that encodes for ARGs and VAGs. Heatmap was used to highlight the distribution of ARGs among the 30 antibiotic classes. The heatmaps were plotted based on the normalized abundance of different antibiotic classes across years, where higher values are shown in red color and white color indicates absence.

## 3. Results

The study analyzed the complete genome of 3963 *E. coli* isolates submitted to the Genome database over a span of more than two decades (Table S1). These strains were reported to be isolated from multiple sources including humans, animals, and plants. However, host information and geographical locations were not available for all the isolates included in this study. Among the 3963 isolates, 3393 had at least one plasmid, with the genome of the *E. coli* strain 2020N21‐110‐15 (accession number: NZ_CM125669.1) containing a maximum of 20 plasmid‐associated sequence records, comprising 10 assembled plasmid replicons and 10 unlocalized plasmid scaffolds.

Violin plot embedded with boxplot analysis of the 3963 genomes showed a median *E. coli* genome size of 5.17 Mb (Figure 2A). The genome size varies between isolates; strain DA33137 (NZ_CP029579.1) had the smallest genome size of 4.45 Mb, whereas strain 48 (NZ_CP166879.1) had the maximum value of 6.25 Mb. Bacterial strains can gain or lose loci or genes, primarily because of horizontal gene transfer mediated by mobile genetic elements, including plasmids. The median G + C content observed was 50.65%. Strain F8111‐1SC3 (NZ_CP024269.1) had the minimum G + C content (50.15%), whereas strain 2022CK‐00450 (NZ_CP104681.1) had the maximum value 51.06%. Previous studies have indicated that G + C content between genome assemblies usually varies by no more than 1% within a given species, and the range (50.15‐51.06%) obtained showed that all the genomes included here belonged to the same organism. All the isolates shared > 96.5% ANI with the reference genome, further supporting their taxonomic ranking as *E. coli*. Overall, the genomes used in this study are unlikely to have significant differences in codon usage, protein structure, and genomic organization.

**Figure 2:**
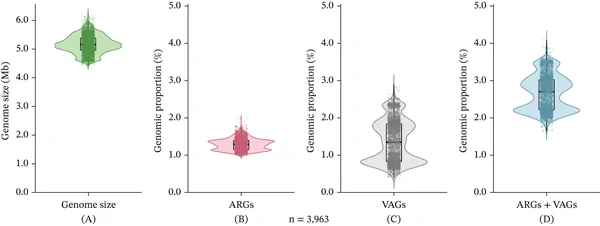
Violin plot embedded with boxplot analysis of genome size of 3963 *E. coli* isolates and percentage of the genome encoded for ARGs and VAGs. (A) Genome size of *E. coli* isolates. (B) Percentage of genome encoded for ARGs. (C) Percentage of genome encoded for VAGs. (D) Percentage of genome encoded for ARGs and VAGs. The *y* axis shows the genome size in Mb, and ARGs and VAGs are presented as percentages.

### 3.1. ARGs

The 2631 ARG reference sequences in the CARD database were used to screen the 3963 *E. coli* genomes. The boxplot analysis showed that the median percentage of *E. coli* genomes encoded for AMR was 1.29%, with a lower quartile of 1.16% and an upper quartile of 1.39% (Figure 2B). A total of 193,713 hits were identified as ARGs across the 3963 genomes, with a median of 47 ARGs per genome. A total of 2037 (51.4%) *E. coli* isolates encoded at least 1.29% of their genome for AMR. Furthermore, the isolates were categorized into four AMR groups on the basis of the percentage of the genome encode for ARGs (Figure 3). Isolates (*n* = 942) in Group I ARGs accounted for 0.98%–1.15% of total genome size. Group II comprised 984 isolates, with ARG content ranging from 1.16% to 1.28% of the genome. Group III, consisting of 961 isolates, encoded 1.29%–1.38% ARGs. Notably, Group IV represented the highest ARG genomic proportion (1.39%–1.99%), and it contains 1076 isolates. Genomes of three isolates has higher share of ARGs, which included strain G3X16‐2 (CP038138.1) with 1.96%, strain GD33 (CP076646.1) with 1.98%, and strain MRSN352231 (CP018103.1) had maximum of 1.99% ARGs. These findings indicate that the proportion of ARG determinants varies across strains, contributing to a spectrum of resistance burdens ranging from minimal to elevated levels.

**Figure 3:**
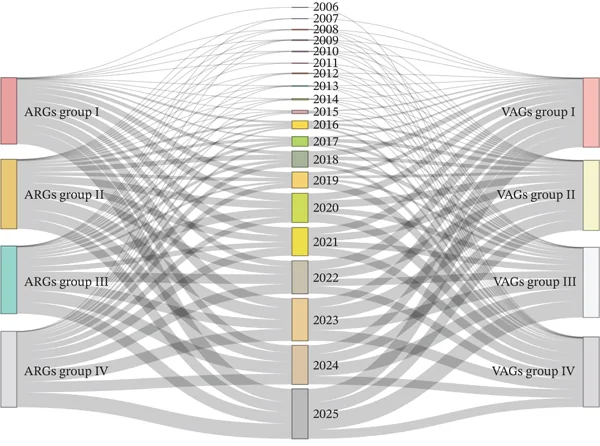
Sankey diagram showing *E. coli* genomes grouped on the basis of the contribution of ARGs and VAGs to overall genome size and the year in which they were submitted the NCBI Genome database. For ARGs, Groups I, II, III, and IV included genomes in which ARGs contributed 0.98%–1.15%, 1.16%–1.28%, 1.29%–1.38%, and 1.39%–1.99% of the overall genome size, respectively. For VAGs, Groups I, II, III, and IV included genomes in which VAGs contributed 0.39%–0.83%, 0.84%–1.34%, 1.35%–1.83%, and 1.84%–2.87% of the overall genome size, respectively. The height of the middle bar is proportional to the ratio of genomes submitted in a specific year.

We identified a total of 293 distinct ARGs in the 3963 *E. coli* isolates. Not all unique ARGs were found in every isolate. However, several isolates contained multiple copies of most of these ARGs. Among these 293 distinct ARGs, 141 were found on both the chromosomes and plasmids, 29 were found only on chromosomes, and 123 were present only on plasmids (Figure 4A; Table S5). The 141 ARGs found on both chromosomes and plasmids suggest that these genes may be transferred between chromosomes and plasmids. Transposition of ARGs into the bacterial chromosome facilitates stable persistence and clonal spread of a resistant bacterial lineage and long‐term retention of resistance loci, even in the absence of plasmids. Genome analyses of the *E. coli* strains UTI89 (NC_007946.1) and APEC 01 (NC_008563.1) submitted in 2006 revealed 41 distinct ARGs, and an additional 252 ARGs were identified from the genomes of the isolates submitted in subsequent years (Table S2). Interestingly, 23 ARGs (*AAC(6*  ^′^ *)-Ib8*, *ACT-7*, *CMY-2*, *CMY-78*, *CTX-M-147*, *CTX-M-25*, *IMP-11*, *IMP-20*, *IMP-8*, *MIR-17*, *MOX-1*, *NmcA*, *OXA-232*, *PDC-1*, *rmtE*, *rmtF*, *SHV-11*, *SHV-2*, *TEM-3*, *TEM-40*, *TEM-79*, *TEM-84*, and *VIM-2*) were found only in the genomes submitted in 2025. This observation further highlights the addition of AMR determinants in *E. coli* strains in the recent past. Previous studies have suggested that ARGs were present in bacteria even before research develops and thus tracing their origin is a complex process.

**Figure 4:**
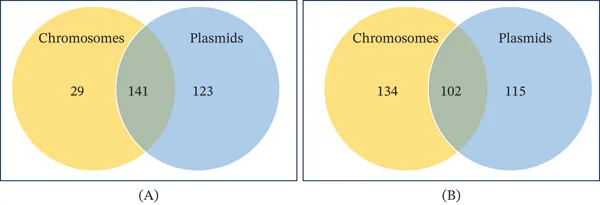
Number of distinct ARGs and VAGs found in the genomes of 3963 *E. coli* isolates. (A) ARG distribution solely on chromosomes and plasmids. (B) VAG distribution solely on chromosomes and plasmids. The ARGs and VAGs present on at least one chromosome or plasmid are shown as common.

The 293 distinct ARGs identified in *E. coli* belonged to 30 drug classes. Among these, 176 ARGs were associated with a single drug class, whereas the remaining 117 ARGs were linked to more than one drug class. Interestingly, *tolC* was associated with 15 classes of drugs, followed by *marA*, which can induce drug resistance against 12 drug classes. Furthermore, the greatest number of ARGs was reported to be associated with the drug class cephalosporin (119), followed by penam (94) and aminoglycoside (56). However, only one ARG was found to be responsible for resistance to drug classes such as benzalkonium chloride (*mdfA*), rhodamine (*mdfA*), and nitroimidazole (*msbA*). In general, antibiotics do not belong to the benzalkonium chloride, rhodamine, or nitroimidazole drug classes, but these contain antibacterial substances, including disinfectants. Heatmap analyses were performed to identify the status of the ARGs and the corresponding drug classes in the *E. coli* isolates. The ARGs identified were found to confer resistance against 30 different classes of antibiotics. It was also observed that over the years, *E. coli* strains have shown greater number of ARGs. The top drug classes based on the abundance of the corresponding ARGs in *E. coli* genomes are fluoroquinolone, penam, tetracycline, cephalosporin, and macrolide (Figure 5). All the genomes analyzed in this study contained at least one ARG from these top five drug classes.

**Figure 5:**
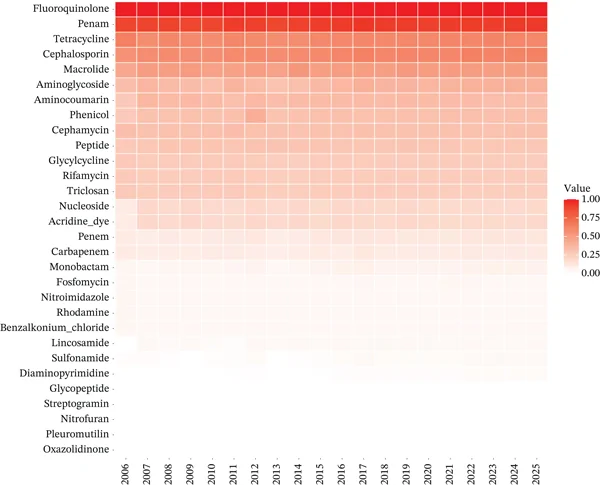
Heatmap of antibiotic resistance genes identified in the genomes of 3963 *E. coli* isolates. The map shows the distribution of ARGs corresponding to the 30 antibiotic classes. The color gradient indicates the relative abundance of ARGs corresponding to each drug class, with darker red representing higher abundance and lighter shades representing lower abundance.

Across 3963 complete *E. coli* genomes, ARG copy numbers varied markedly between genome assemblies (Table S6). The strain MRSN352231 (Acc. No. NZ_CP018103.1) showed the highest total of ARG hits (*n* = 89), with 35 copies of *TEM-57* (which was the most amplified single ARG in the dataset), along with *tet(A)* (two copies) and *CTX-M-15* (1 copy). Increase in the number of ARGs or VAGs may increase resistance and bacterial survival. In terms of diversity of ARGs, the genome of strain GD33 (NZ_CP076646.1) encoded the highest number of unique ARGs (*n* = 74) out of 88 copies, featuring three copies each of *floR* and *tet(A)* and two copies each of *aadA2*, *TEM-1*, *OXA-10*, and *dfrA14*. This strain was reported as multidrug‐resistant and was isolated from poultry. Furthermore, ≥ five copies of 26 ARGs were found in at least one strain, and *TEM-1* was the most prevalent ARG detected in 1326/3963 genomes (33.5%), of which 258 genomes contained two or more copies, with a maximum of 12 copies found in strain KE47 (NZ_CP141089). Other key ARGs had similar copy number gain patterns, including *tet(A)* which was found in 1340 isolates (maximum six copies), *sul1* (1127 genomes [28.4%], maximum seven copies), *mphA* (1000 genomes [25.2%], maximum eight copies), and *CTX-M-15* (476 genomes [12.0%], maximum six copies). These observations indicate that gene amplification, particularly of the *β*‐lactamase families such as *TEM*, increases the resistome in some isolates beyond the simple qualitative, presence–absence reports. Interestingly, single copy of *CRP* and *cpxA* were found in all 3963 genomes (100% prevalence), and these ARGs may represent conserved regulatory genes with reported roles in modulating antimicrobial susceptibility. In addition, several near‐core genes (found in > 99% isolates) were identified which included the efflux components (*acrA*, *acrB*, *tolC*, *mdfA*, and *msbA*) and global regulators (*marA* and *H-NS*). These findings indicate a stable intrinsic multidrug efflux and regulatory system in *E. coli.*

We observed that ≥ 1.75% of the genome encodes for AMR determinants across 17 analyzed strains, highlighting a substantial genomic investment for resistance mechanisms. Comprehensive literature screening also showed that seven strains—CRE1540, EC11, GD33, JNQH497, MRSN352231, NDM5, and WCHEC025943—have previously been reported as antimicrobial resistant, supporting the notion that a higher percentage of genomic regions encoding for ARGs may induce multidrug resistance. Notably, five of these strains (MRSN352231, CRE1540, EC11, GD33, and NDM5) were found to be harboring 73–89 copies of ARGs, indicating a high number of resistance determinants. The elevated ARG copy numbers observed in these strains suggest extensive acquisition and potential accumulation of resistance elements, which may contribute to multidrug‐resistant phenotypes. Overall, the substantial proportion of AMR encoding genomic regions, combined with the high ARG copy numbers in previously reported resistant strains, underscores the significant role of genomic plasticity and gene transfer that may increase the spread of antimicrobial resistant strains. These findings reinforce the need for continuous genomic surveillance to monitor the dissemination and evolution of resistance determinants.

### 3.2. VAGs

It was observed that the number of VAGs and their abundance are relatively greater than the number of ARGs and their abundance in the genomes of *E. coli* isolates. The median percentage of the *E. coli* genome that represented VAGs was estimated to be 1.35%, with a lower quartile of 0.84% and an upper quartile of 1.83% (Figure 2C; Table S3). Out of the 3963 *E. coli* isolates compared, 1987 (50.13%) VAGs constituted at least 1.35% of the genomes. The *E. coli* genomes were categorized into four groups based on the prevalence of VAGs (Figure 3). Isolates (*n* = 983) in Group I encoded 0.39%–0.83% of their genomes for VAGs. Group II comprised 993 isolates, with VAG content ranging from 0.84% to 1.34% of the genome. Group III consisted of 996 isolates, and 1.35%–1.83% of their genome encode for VAGs. Notably, Group IV represented the highest VAG burden (1.84%–2.87%) and this group contains 991 isolates. These findings indicate that the proportion of VAG determinants varies across strains that may contribute to resistance.

A total of 351 distinct VAGs were identified in the *E. coli* genomes. Among these, 102 were found on both the chromosomes and plasmids, 134 were found only on the chromosomes, and 115 were present only on the plasmids (Figure 4B; Table S5). A total of 92 distinct VAGs were identified in the genomes of the *E. coli* strains UTI89 (NC_007946.1) and APEC 01 (NC_008563.1) submitted in 2006. An additional 259 VAGs were identified in the genomes submitted in subsequent years (Table S4). The copy number of VAGs varied substantially among the *E. coli* genomes. The genome of *E. coli* strain EDL933 (NZ_CP124740.1) had the highest number of VAGs (*n* = 122), whereas the genome of strain DEC5E (NZ_CP038383.1) had the highest number of distinct VAGs (*n* = 120). This observation highlights the variability in the virulence gene repertoire and diversity among *E. coli* strains. Gene amplification was observed for some loci. For example, strain TUM1886 (NZ_CP128970.1) had 20 copies of the *astA* gene, and this represents the highest amplification for any single VAG. Such VAG amplification may contribute to strain‐specific enhancement of virulence. Notably, there was no virulence gene set found commonly in all isolates, indicating that virulence determinants in *E. coli* genomes are an accessory gene set.

### 3.3. ARG–VAG Distribution in STs

MLST assigned 3879 genomes to 632 STs, whereas 84 genomes could not be assigned a valid ST. The most frequently detected lineage was ST131 (*n* = 411), followed by ST10 (*n* = 364), ST11 (*n* = 275), ST410 (*n* = 126), and ST167 (*n* = 112) (Figures 6A,B). The distribution of ARG groups among the 20 most prevalent STs is shown in Figure 6C. ST11 (*n* = 210) was most frequently represented in ARG Group I, whereas ST131 (*n* = 193) predominated in ARG Group II. Interestingly, ST10 was the most frequently represented ST in both ARG Groups III (*n* = 161) and IV (*n* = 162). ST1060 (*n* = 47) harbored a higher level of ARGs, with 76.59% of isolates carrying Group IV ARGs and the remaining 23.41% carrying Group III ARGs. Similarly, VAG groups among the 20 most prevalent STs are presented in Figure 6D. ST10 was the most frequently represented lineage in VAG Groups I (*n* = 267) and II (*n* = 71). However, ST131 (*n* = 267) was most frequently represented in VAG Group III, whereas ST11 (*n* = 274) was abundant in VAG Group IV. Interestingly, almost all the strains in ST11 (*n* = 274/275) and ST73 (*n* = 57) were found to carry group IV virulence genes. Overall, these findings demonstrate a lineage‐specific distribution of AMR and virulence determinants among the predominant *E. coli* STs. The dominance of ST131, ST10, and ST11 across different ARG and VAG groups highlights their potential importance in the dissemination of resistance and virulence traits. In particular, the representation of ST10 across multiple ARG and VAG groups suggests a genetically diverse lineage, whereas ST131 and ST11 appear to carry distinct resistance–virulence profiles. These high‐frequency lineages may therefore play an important role in the persistence and spread of antimicrobial resistant *E. coli* strains.

**Figure 6:**
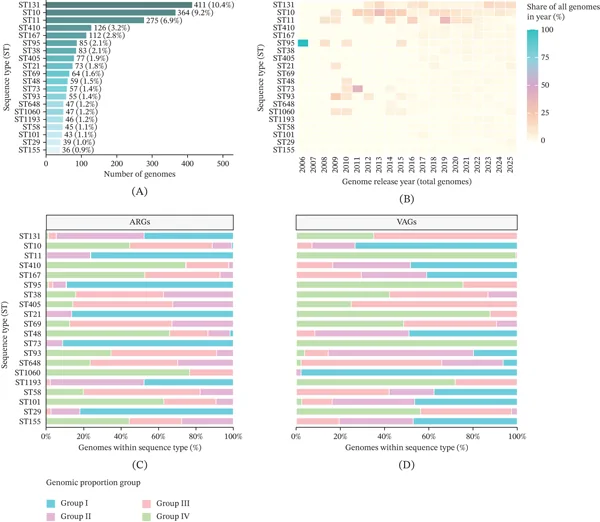
Top 20 *E. coli* sequence types (STs) identified from 3963 genomes. (A) Most abundant STs in *E. coli* isolates. (B) Year‐wise distribution of STs. (C) Genomic proportion of ARGs. (D) Genomic proportion of VAGs.

### 3.4. Temporal Trend Analysis of ARGs and VAGs

We conducted a comprehensive trend analysis of *E. coli* isolates encoding ARGs and VAGs. This analysis is aimed at assessing temporal variation in ARGs and VAGs genomic profiles across genome release over the years. The overall nonparametric Mann–Kendall trend and weighted linear regression analysis report is provided in Table 1 and Figure 7. The temporal analysis revealed a modest but statistically significant increase in the genomic proportion of ARGs among *E. coli* genomes submitted between 2006 and 2025. This finding was supported by the Mann–Kendall test (*τ* = 0.463, *p*‐value  = 0.0048), whereas Sen′s slope analysis indicated an increase of 0.00433% ARGs per year (95% CI: 0.00213–0.00716). Weighted linear regression also showed 0.00292 percentage increase of ARGs per year in *E. coli* genomes (95% CI: 0.00075–0.00509; *p* value = 0.0111). Together, these results indicate that the ARG burden of the sampled *E. coli* genomes tended to increase over the study period.

**Table 1:** Temporal trends of ARGs and VAGs in 3963 *Escherichia coli* genomes released over a period of 2006–2025.

Measure	Mann–Kendall *τ*	MK *p*	Sen′s slope	Sen′s slope 95% CI	Weighted regression *β*	Regression *β* 95% CI	Weighted *R* ^2^	Weighted regression *p*
ARG	0.463	0.0048	0.00433	0.00213–0.00716	0.00292	0.00075–0.00509	0.308	0.0111
VAG	−0.189	0.2561	−0.00561	−0.02380 to 0.00831	−0.00712	−0.01967 to 0.00544	0.073	0.2493
ARG + VAG	−0.063	0.7212	−0.00184	−0.01587 to 0.00870	−0.00419	−0.01577 to 0.00739	0.031	0.4567

*Note:* Temporal trends were predicted using mean genomic region encode for ARGs and VAGs per year. Mann–Kendall analysis was used to assess monotonic trends, and Sen′s slope was used to estimate the percentage gain or loss over the period. Weighted regression analysis was performed to avoid unequal sample size per year.

**Figure 7:**
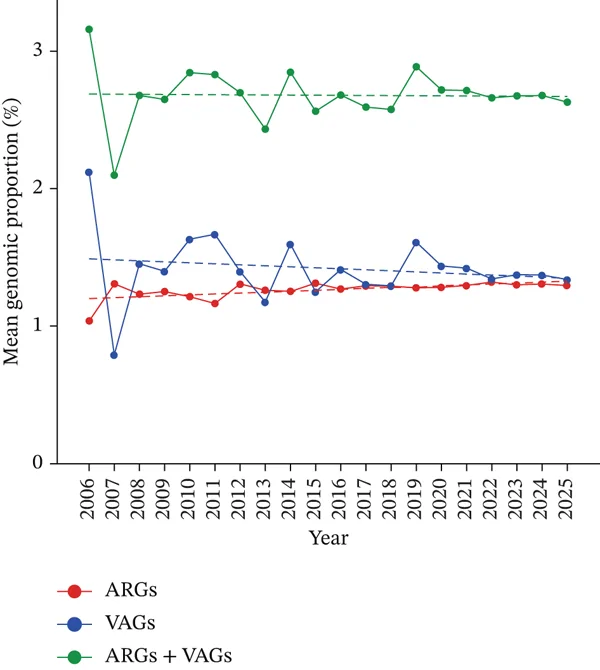
Overall trend analysis of ARGs and VAGs in the genomes of 3963 *E. coli* isolates. The dotted line represents the overall linear trend over time.

In contrast, no statistically significant temporal trend was detected for VAG proportion in *E. coli* genomes submitted between 2006 and 2025. The Mann–Kendall test showed a weak negative association (*τ* = −0.189, *p* value = 0.2561), and Sen′s slope was −0.00561% per year (95% CI: −0.02380 to 0.00831). Similarly, weighted linear regression also showed no significant temporal decrease (*β* = −0.00712 percentage points per year, 95% CI: −0.01967 to 0.00544, *p* value = 0.2493; *R* ^2^ = 0.073). The median percentage of the *E. coli* genome that contained ARG and VAGs was estimated to be 2.7%, with a lower quartile of 2.23% and an upper quartile of 3.03% (Figure 2D). The combined ARG–VAG genomic proportion showed no statistically significant temporal trend (*τ* = −0.063, *p* value = 0.7212), and Sen′s slope of −0.00184 percentage decrease per year (95% CI: −0.01587 to 0.00870). Weighted linear regression also showed temporal decrease over the years (−0.00419%), but the combined ARG–VAG decrease was not statistically significant (95% CI: −0.01577 to 0.00739, *p* value = 0.4567; *R* ^2^ = 0.031). Overall, the findings provide evidence of a gradual increase in genome that encodes for ARGs in *E. coli* isolates, whereas the genomic contribution of VAGs remained comparatively stable. This trend may reflect increasing antimicrobial selection pressure, and changes in the prevalence of resistant lineages.

## 4. Discussion

Comprehensive profiling of AMR and virulence gene carriage in 3963 *E. coli* genomes identified 293 distinct ARGs and 351 distinct VAGs, and their distinct distribution in chromosomes and plasmids, with substantial variation in gene copy number and differing temporal patterns for ARG and VAG genomic proportions. Bacteria often exhibit diverse mechanisms that contribute to the spread of antibiotic resistance. ARGs are capable of introducing structural changes to a drug molecule that constitute a well‐known mechanism of acquired antibiotic resistance in bacterial species. Previous studies have shown that ARG carrying prophage acquisition confers a fitness advantage to bacteria under antibiotic exposure [31]. Antibiotic‐resistant *E. coli* and other bacterial pathogens remain a major burden in immunocompromised humans and animals [32]. The availability of 1000s of whole‐genome sequences of bacterial pathogens has made comparative studies of AMR and virulence much more meaningful. In this study, *E. coli* isolates were found to have genome sizes that ranged from 4.45–6.25 Mb, and this could be due to the gain and loss of genes and loci during adaptation. It has been reported that bacterial genome size increases because of the acquisition of genes in response to new selection pressures in the environment [33, 34].

Comparative analyses of the *E. coli* genomes (*n* = 3963) produced 293 best‐hit ARGs which are known to confer resistance to 30 different classes of antibiotics. The *E. coli* genome submitted in 2006 contained 41 different ARGs. Several reports have indicated that some ARGs are ancient and that they became more common after the use of antibiotics became a standard practice [34, 35]. Development of AMR can be due to either mutation of preexisting genes or the acquisition of genes through horizontal gene transfer of ARGs [36]. Bacterial strains can contain single or multiple ARGs, and the presence of multiple ARGs in a strain may contribute to overall AMR. However, it has been reported that the number alone does not necessarily determine the phenotypic level of AMR [37].

Plasmids play a crucial role in the spread of antibiotic resistance because they facilitate horizontal gene transfer between bacterial species. The identification of 123 plasmid‐borne ARGs along with 141 ARGs on both chromosomes and plasmids indicates the role of plasmids in the spread of AMR. The role of plasmids in virulence is bolstered by the identification of 115 plasmid‐borne VAGs along with 102 VAGs on both chromosomes and plasmids. In a recent study, 2420 complete bacterial genomes were compared, and it was reported that 87% of the ARGs have the potential to be transferred among different plasmids [38]. Another study revealed that ARGs were colocalized on chromosomes and/or large plasmids, further corroborating the role of plasmids in the transfer of ARGs [39]. It has been reported that there is a positive association between ARGs and VAGs in *E. coli* isolates from clinically healthy waterfowl [40], suggesting the need to screen for VAGs in environmental samples.

Network analysis showed that the maximum number of ARGs (119) targeted the cephalosporin drug class, followed by penam (94) and aminoglycoside (56). Previous studies have shown that *E. coli* strains containing multiple ARGs have a higher level of resistance than those harboring a single ARG [41]. Another study indicated that *E. coli* strains containing *tetD* were sensitive to tetracycline, whereas those containing *tetA* and/or *tetB* were resistant to tetracycline [42]. Heatmap analysis showed that fluoroquinolone, penam, tetracycline, cephalosporin, and macrolide were the top five drug classes in relation to the number of ARGs identified. The predominance of ARGs associated with these drug classes highlights the extensive genetic basis of resistance to commonly used antimicrobials and underscores the importance of continued genomic surveillance to monitor the emergence and dissemination of resistance determinants. Over the years, *E. coli* strains seem to have multiple ARGs that confer protection against several antibiotics. A study on the infant gut resistome revealed that *E. coli* ARGs corresponding to the drug classes of tetracycline and fluoroquinolone were by far the most common, followed by those corresponding to penam, cephalosporin, macrolides, and cephamycin [43]. Analysis of 14,548 episodes of bloodstream infections due to *E. coli* revealed that resistance against aminopenicillins was the most common, followed by resistance against cephalosporins, fluoroquinolones, sulfonamides, cephalosporins, aminoglycosides, beta‐lactam‐beta‐lactamase‐inhibitors, and carbapenems [44].

Parametric and nonparametric trend analysis revealed a slight increase in the percentage of *E. coli* genomes containing ARGs. Although the ARGs were found mostly on plasmids, genes encoding toxins were associated primarily with chromosomes. It has been reported that ARGs are usually located on plasmids, which contribute to their spread among different bacteria [45]. Furthermore, plasmids can exchange genetic material with chromosomes and other plasmids and can thus contribute to the spread of AMR [46]. However, the exact mechanism of transfer of ARGs between different genetic elements remains unknown. A recent meta‐analysis reported that bacterial cells are impacted more by the accumulation of mutations in genes that confer antibiotic resistance than by the acquisition of ARGs [47]. Overall, results of the current study indicate the necessity for the development of alternative approaches to overcome the burden of AMR and virulence. In 17 strains, 1.75%–1.95% of the genome was found to encode for ARGs, indicating an increased number of ARGs that may contribute resistance mechanisms in some strains. Literature review showed that seven strains—CRE1540 [48], EC11 [49], GD33 [50], JNQH497 [51], MRSN352231 [52], NDM5 [53], WCHEC025943 [54]—were previously reported to be resistant to antibiotics. However, it has to be noted that the presence of ARGs alone may not contribute to resistance, as other mechanisms may also contribute to the resistance.

The virulence potential of *E. coli* is determined by a diverse repertoire of VAGs that facilitate colonization, adhesion, invasion, nutrient acquisition, immune evasion, and persistence within the host. In the present study, the number and diversity of VAGs detected across *E. coli* strains were substantially greater than those of ARGs, indicating their genomic capacity to encode multiple mechanisms associated with host interaction and pathogenicity and emphasizes the importance of considering virulence determinants alongside AMR. A slightly greater unique number of virulence genes was found to be located solely on chromosomes than exclusively on plasmids or shared between both. The temporal trend analysis showed slight decrease in carriage of virulence determinants across the genomes over the years, but this shift was not significant.

ST analysis revealed that *E. coli* ST10 was the most frequently represented ST in both ARG Groups III and IV. Consistent with this finding, previous studies have reported that more than 71% of ST10 isolates exhibit multidrug resistance [55]. Similarly, nearly all isolates belonging to ST11 and ST73 harbored Group IV virulence genes. Previous studies have also identified ST11 [56] and ST73 [57] as highly virulent *E. coli* lineages. Weighted linear regression analysis of temporal trends further demonstrated a 0.00292 percentage increase in the proportion of *E. coli* genomes encoding ARGs. This temporal increase may reflect the ongoing acquisition and maintenance of ARGs, potentially driven by fitness advantages conferred through horizontal gene transfer, as previously reported in *E. coli* [58].

One of the limitations of this study is the use of genomic data from public repositories which do not solely represent community isolates and might overrepresent hospital outbreaks. This study analyzed only the complete genome assemblies available at the public databases. Metadata including geographic origin, host source, clinical context, and antibiotic exposure history were incomplete for many isolates, limiting detailed epidemiological stratification. Although gene presence and copy number were comprehensively assessed, the analysis did not incorporate gene expression profiling, phenotypic resistance testing, or functional validation. Therefore, rather than reflecting phenotypic features that have been experimentally proven, the data highlight genetic potential for virulence and antibiotic resistance.

## 5. Conclusions

This study presents a comprehensive analysis of 3963 *E. coli* genomes in the context of ARGs and VAGs. A total of 293 distinct ARGs and 351 distinct VAGs were identified from the genomes of *E. coli* isolates. The analysis identified that in 51.4% of the isolates, ARGs accounted for at least 1.29% of their genome, whereas in 50.13% of the isolates, virulence genes contributed to at least 1.35% of their genome. Exchange of ARGs from plasmids to chromosomes may increase the spread of AMR strains. ST analysis showed that ST10 was the most frequently represented in both higher level ARG Group III and IV strains, and almost all the strains in ST11 and ST73 were found to carry Group IV virulence genes. Weighted linear regression trend analyses showed a 0.00292 percentage increase in genome per year encoding for ARGs in *E. coli*, whereas the slight decrease of VAGs observed was not significant. Genome‐wide analyses have the potential to enhance our understanding of AMR and may contribute to improving the strategies to combat infectious diseases. Array of genes that confer resistance against common antimicrobials suggests the need for novel strategies to combat AMR.

## Author Contributions

D.S. analyzed the data, plotted the graphs, and drafted the manuscript. A.S.T., A.S., I.T., A.B.K., and A.D.M. performed the statistical analysis. T.S.M. and S.S. reviewed the data and analyses and edited the manuscript. B.P. conceptualized, supervised the work, and edited the manuscript.

## Funding

This study was supported by the Manipal Academy of Higher Education (MAHE), Manipal, for the publication charges.

## Disclosure

All authors read and approved the final version of the manuscript.

## Ethics Statement

The authors have nothing to report.

## Conflicts of Interest

The authors declare no conflicts of interest.

## Supporting information


**Supporting Information** Additional supporting information can be found online in the Supporting Information section. Accession numbers of 3963 genomes used in this study are provided in the supplementary files. Table S1: Prevalence of ARGs. Table S2: Distinct ARGs over the years. Table S3: Prevalence of VAGs. Table S4: Distinct VAGs over the years. Table S5: Distinct ARGs and VAGs in chromosomes and plasmids. Table S6: Copy number of ARGs.

## Data Availability

The data that supports the findings of this study are available in the supplementary material of this article.
